# Predicting factors of central lymph node metastasis and *BRAF*^*V600E*^ mutation in Chinese population with papillary thyroid carcinoma

**DOI:** 10.1186/s12957-021-02326-y

**Published:** 2021-07-13

**Authors:** Sheng Li Zhou, Yan Ping Guo, Lei Zhang, Tao Deng, Zi Guang Xu, Chao Ding, Wen Cong Sun, Yue Wu Zhao, Ling Fei Kong

**Affiliations:** 1 Department of Pathology, Henan Provincial People’s Hospital, People’s Hospital of Zhengzhou University, People’s Hospital of Henan University, 7# Weiwu Road, Zhengzhou, 450003 Henan Province China; 2 Department of Thyroid Surgery, Henan Provincial People’s Hospital, People’s Hospital of Zhengzhou University, People’s Hospital of Henan University, Zhengzhou, 450003 Henan Province China

**Keywords:** Papillary thyroid carcinoma, *BRAF*^*V600E*^ mutation, Tumor size, Central lymph node metastasis

## Abstract

**Objective:**

The aim of this study was to evaluate the predictive factors of central lymph node metastasis (CLNM) and *BRAF*^*V600E*^ mutation in Chinese patients with papillary thyroid carcinoma (PTC).

**Methods:**

A total of 943 PTC patients who underwent thyroidectomy from 2014 to 2016 at our hospital were enrolled. Those patients were divided into PTC > 10 mm and papillary thyroid microcarcinoma (PTMC) groups by tumor size. The *BRAF*^*V600E*^ mutation was examined by quantitative real-time PCR. Univariate and multivariate analyses were used to examine risk factors associated with CLNM and the *BRAF*^*V600E*^ mutation.

**Results:**

The frequency of CLNM was 53% (505/943). Both univariate and multivariate analyses suggested that the risk factors for CLNM in PTC patients were male, younger age, and larger tumor size (*P <* 0.05). Coexistent Hashimoto thyroiditis (HT) was an independent protective factor against CLNM when the tumor was > 10 mm (*P =* 0.006). Stratified analysis revealed that male, age ≤ 30 years, and tumor size > 5 mm were independent risk factors for CLNM. The *BRAF*^*V600E*^ mutation rate was 85%. Multivariate logistic regression analysis revealed that age (*P <* 0.001) and coexistent HT (*P* = 0.005) were independent predictive factors of *BRAF*^*V600E*^ mutation in PTC patients. Only age was a risk factor for the *BRAF*^*V600E*^ mutation when the tumor was > 10 mm (*P* = 0.004). In the PTMC group, the *BRAF*^*V600E*^ mutation was significantly correlated with tumor size (*P* < 0.001) and coexistent HT (*P* = 0.03). Stratified analysis revealed that age > 30 years and tumor size > 5 mm were independent predictive factors of *BRAF*^*V600E*^ mutation. Furthermore, the incidence of CLNM was significantly higher in *BRAF*^*V600E*^ mutation-positive patients (*P* = 0.009) when the tumor was ≤ 5 mm.

**Conclusion:**

The factors male, younger age (≤ 30 years), large tumor size (> 5 mm), and coexistent HT are independent predicative factors for CLNM. The *BRAF*^*V600E*^ mutation is associated with both large size and without HT in PTMC patients, age > 30 years in the PTC > 10 mm group. The *BRAF*^*V600E*^ mutation was an independent risk factor for CLNM when the tumor was ≤ 5 mm. For optimal management, these features should be comprehensively evaluated to determine the initial surgical approach for PTC patients.

## Introduction

The global incidence of thyroid cancer is rapidly increasing at a remarkable rate [1, 2]; currently, the incidence of thyroid cancer ranks ninth among all cancers. This increase in incidence is mostly attributed to papillary thyroid cancer (PTC), in particular papillary thyroid microcarcinoma (PTMC), which has a maximum diameter of 1 cm or less [2, 3]. Currently, the widespread use and technical improvement of ultrasound (US) and US-guided fine-needle aspiration biopsy (FNAB) have increased the detection rate of PTMC [4].

It is known that PTC patients, especially PTMC patients, have an excellent prognosis. However, central lymph node metastasis (CLNM) in PTC patients, even in those with PTMC, is not uncommon. The detection rate of CLNM ranges from 13.4 to 64% among PTC patients [5–9]. Lymph node metastasis is an important risk factor for cancer progression and recurrence. Therefore, early detection of CLNM plays an important role in choosing therapeutic strategies. To date, several researchers have identified possible predictive clinicopathological risk factors for CLNM, and the results of these studies are inconsistent [10]. Thus, it is necessary to identify clinicopathological factors associated with CLNM in PTC patients to guide therapeutic decisions.

With the emerging understanding of molecular genetics in thyroid cancer, several specific molecular events in PTC have been identified that assist in the diagnosis and prognostic evaluation of PTC patients [11–13]. The *B-type Raf kinase* (*BRAF*) ^*V600E*^ mutation was found to be the most important candidate biomarker for PTC diagnosis because of its high prevalence and high specificity [14, 15]. The *BRAF*^*V600E*^ mutation abnormally activates the mitogen-activated protein kinase pathway and increases cell proliferation and differentiation, eventually resulting in tumorigenesis [14]. The prevalence of the *BRAF*^*V600E*^ mutation ranges from 33.2 to 88% [15]. However, the relationship between the *BRAF*^*V600E*^ mutation and specific clinicopathological features of PTC remains controversial. Therefore, it is important to discriminate the features related to the *BRAF*^*V600E*^ mutation in PTC patients.

Thus, the purpose of this study was to evaluate the relationship between clinicopathological features and CLNM and the *BRAF*^*V600E*^ mutation status to obtain information necessary to optimize therapeutic decisions for PTC patients.

## Patients and methods

### Patients

All PTC patients in the present study who underwent either total thyroidectomy or near-total thyroidectomy in Henan Provincial People’s Hospital between October 2014 and October 2016. The medical records of these patients were retrospectively analyzed. The histopathlogic examinations of all patient were performed by two experienced pathologists independently and a consensus was made blinded to *BRAF*^*V600E*^ status. Only conventional PTC that with papillae structure and PTC-type nuclei features were enrolled in the present study. In total, 943 patients were recruited for this study [men: 233, mean age(average ± standard deviation), 52.0 ± 11.6 years; women: 710, mean age, 45.4 ± 11.6 years]. None of these patients received radioactive iodine^131^ therapy before surgery. The preoperative levels of free triiothyronine, free thyroxine, thyroid-stimulating hormone, thyroglobulin antibodies, and thyroid peroxidase antibodies were detected by radioimmunoassay. Hashimoto’s thyroiditis (HT) was confirmed based on the postoperative pathologic findings and an elevated preoperative serum thyroid autoantibody level. Clinicopathologic staging was classified based on the 8th edition of the tumor-node-metastasis (TNM) staging published by the American Joint Committee on Cancer. The mean follow-up duration was 27 postoperative months (range = 11–44 months). At the time of follow-up, only one patient died of breast cancer.

### Analyses of the *BRAF*^*V600E*^ mutation

Genomic DNA isolated from paraffin-embedded tissues of PTC patients. The paraffin-embedded tissue blocks of tumor tissue was sectioned for hematoxylin and eosin staining with the purpose of identifying pathological diagnosis. Regions with neoplastic compositions of 80% or greater were marked as tumor tissue under optical microscope. Depending on the size of the tissue, 3–4 pieces of 5-μm-thick sections of each block were manually microdissected using a needle and collected into 1.5 ml microtubes for DNA extraction. The Genomic DNA isolation process was performed using the Puregene Tissue Kit (Qiagen, Valencia, CA, USA), according to the manufacturer’s instructions. The absorbance of the DNA samples was measured using a spectrophotometer, and only the A260/A280 values between 1.8 and 2.0 were accepted for the next step. The extracted DNA was stored at – 80 °C for next procedure. The status of the *BRAF*^*V600E*^ mutation of each sample was examined by qRT-PCR using the AmoyDx *BRAF*^*V600E*^ Mutation Detection Kit (Amoy Diagnostics, Xiamen, China), and the presence of the mutation was evaluated following the manufacturer’s instructions. The sample was classified as mutation-positive when the cycle threshold (*Ct*) was ≤ 28 and as negative when *Ct* was > 28.

### Statistical analyses

All statistical analyses in the present study were conducted using SPSS 21 (IBM Corporation, Waltham, NY, USA). Enumeration data were presented as frequencies and were compared utilizing the chi-squared or Fisher’s exact test. Measurement data were presented as means ± standard deviation and using the independent sample *t* test for variables for normal distribution and Mann-Whitney for non-normal distribution. The effects of the clinicopathologic factors on central lymph node metastasis (CLNM) and *BRAF*^*V600E*^ mutation were evaluated using univariate and multivariate logistic-regressions. For all test, a probability value of < 0.05 was considered statistically significant.

## Results

### Clinicopathologic characteristics of patients with PTC

The clinical and pathological characteristics of the 943 patients are shown in Table 1. There were 233 (25%) male patients and 710 (75%) female patients, and the male-to-female ratio was 1:3.0. The age of the patients ranged from 15 to 77years, with an average age of 45.9 ± 11.3 years. The tumor size ranged from 1 to 50 mm, and the average size was 1.2 ± 0.9 mm. There were 688 patients with unifocality (73%) and 255 patients with multifocality (*n* ≥ 2) (27%). HT was rare among the PTC patients (5%, 50/943). CLNM was common among the PTC patients, with a frequency of 53% (504/943). The proportions of patients diagnosed with stage T1, T2, and T3 disease were 89% (838/924), 8% (80/943), and 3% (25/943), respectively. In total, there were 856 (91%) patients in TNM stage I and 87 (9%) patients in stage II. The *BRAF*^*V600E*^ mutation rate was 85% (806/943).

**Table 1:** The correlation of tumor size with clinicopathologic characteristics of 943 patients with papillary thyroid carcinoma between 2014 and 2016

Variable	Overall PTC (*n* = 943)		PTC > 10 mm (*n* = 373)		PTMC (*n* = 570)		Univariate analysis	Multivariate analysis	
	*n*	(%)	*n*	(%)	*n*	(%)	*P*	*P*	OR(95CI)
Gender									
Male	233	(25)	107	(29)	126	(22)	**0.02**	0.899	0.98(0.70–1.38)
Female	710	(75)	266	(71)	444	(78)			
Age (years) ^*^	(45.9 ± 11.3)		(45.4 ± 12.0)		(46.3 ± 10.8)		0.265		
Focality									
Uni	588	(73)	259	(69)	429	(75)	0.05	0.178	1.25(0.90–1.73)
Multi	255	(27)	114	(31)	141	(25)			
HT									
Yes	X50	(5)	15	(4)	35	(6)	0.159		
No	893	(95)	358	(96)	535	(94)			
CLNM									
Yes	505	(53)	275	(74)	230	(40)	**< 0.001**	**<0.001**	4.10(3.04–5.53)
No	438	(47)	98	(26)	340	(60)			
TNM staging									
I	856	(91)	320	(86)	536	(94)	**< 0.001**	0.554	1.19(0.66–2.15)
II	87	(9)	53	(14)	34	(6)			
*BRAF*^*V600E*^ mutation									
Yes	806	(85)	311	(83)	495	(87)	0.141		
No	137	(15)	62	(17)	75	(13)			

*Abbreviations*: *PTC* papillary thyroid carcinoma, *PTMC* papillary thyroid microcarcinoma, *HT* Hashimoto’s thyroiditis, *CLNM* central lymph node metastasis

*Numbers are presented as means ± standard deviation

Of the 943 PTC patients, there were 373 (40%) patients with PTC > 10 mm and 570 (60%) PTMC patients (Table 1). The average tumor sizes were 2.0 ± 1.0 mm and 0.6 ± 0.2 mm, respectively (*P* < 0.001). The univariate analysis revealed that there were more male patients (*P =* 0.02), more patients with CLNM (*P* < 0.001), and higher TNM stages (*P* < 0.001) in the PTC > 10 mm group than in the PTMC group. The multivariate analysis revealed that only CLNM was significantly correlated with PTC > 10 mm [*P* < 0.001, odds ratio (OR) = 4.10, 95% confidence interval (95% CI) 3.04–5.53]. Even though the frequency of the *BRAF*^*V600E*^ mutation was slightly higher in the PTMC group (87%) than in the PTC > 10 mm group (83%), there was no statistically significant difference (*P =* 0.074). No difference was found in age, tumor size, or HT between the two groups.

### The relationships between CLNM and the clinicopathological characteristics of PTC patients

The relationships between CLNM and the clinicopathological characteristics of the PTC patients are shown in Table 2. Both univariate and multivariate analyses revealed that the risk for CLNM was significantly associated with male, younger age, and larger tumor size among PTC patients, regardless of the tumor size (*P <* 0.05). In addition, in both the overall and PTC > 10 mm groups, the positive rate of CLNM was significantly decreased in PTC patients with coexistent HT compared with patients without HT, which suggested that coexistent HT might be a protective factor against CLNM in the PTC patients (*P* = 0.002). Furthermore, advanced T stage was also a risk factor for CLNM in the PTC patients (*P* < 0.001, OR = 4.94, 95% CI 2.84–8.59).

**Table 2:** Relationships between central lymph node metastasis mutation and clinicopathologic characteristics of 943 patients with PTC

Variable	Overall PTC(*n* = 943)					PTC > 10 mm(*n* = 373)					PTMC(*n* = 570)				
	Yes	No	Uni- and multivariate analysis			Yes	No	Uni- and multivariate analysis			Yes	No	Uni- and multivariate analysis		
	*n*(%)	*n*(%)	*P*	*P*	OR(95CI)	*n*(%)	*n*(%)	*P*	*P*	OR(95CI)	*n*(%)	*n*(%)	*P*	*P*	OR(95CI)
Gender															
Male	166(71)	67(29)	**< 0.001**	**< 0.001**	0.41 (0.28–0.58)	90(84)	17(16)	**0.004**	**0.02**	0.48 (0.26–0.88)	76(60)	50(40)	**< 0.001**	**< 0.001**	0.37 (0.24–0.57)
Female	339(48)	371(52)				185(70)	81(30)				154(35)	290(65)			
Age (years) ^*^	43.6 ± 12.2	47.2 ± 9.8	**< 0.001**	**< 0.001**	0.97 (0.96–0.98)	43.4 ± 12.6	49.2 ± 9.1	**< 0.001**	**< 0.001**	0.96 (0.94–0.98)	43.9 ± 11.7	46.6 ± 10.0	**0.001**	**0.01**	0.98 (0.96–0.99)
Tumor size (mm)^*^	1.5 ± 1.1	0.9 ± 0.7	**< 0.001**	**< 0.001**	3.45 (2.48–4.79)	2.1 ± 0.8	1.8 ± 0.8	**0.01**	**0.02**	1.49 (1.08–2.06)	0.7 ± 0.2	0.6 ± 0.2	**0.002**	**0.002**	3.65 (1.62–8.21)
Focality															
Uni	355(52)	333(48)	0.055			186(72)	73(28)	0.206			169(39)	260(61)	0.417		
Multi	150(59)	105(41)				89(78)	25(22)				61(43)	80(57)			
HT															
No	491(55)	402(45)	**< 0.001**	**0.03**	0.42 (0.19–0.91)	270(75)	88(25)	**< 0.001**	**0.006**	0.21 (0.07–0.64)	221(41)	314(59)	0.068		
Yes	14(28)	36(72)				5(33)	10(67)				9(26)	26(74)			
T staging															
T1	418(50)	420(50)	**< 0.001**	**< 0.001**	4.94 (2.84–8.59)	188(70)	80(30)	**0.01**	0.723		230(40)	340(60)	–		
T2 + T3	87(83)	18(17)				87(83)	18(17)				0	0			
*BRAF*^*V600E*^ mutation															
No	75(62)	62(38)	0.742			51(82)	11(17)	0.095			25(40)	50(60)	0.121		
Yes	430(36)	376(64)				224(72)	87(28)				205(41)	290(59)			

*Abbreviations*: *PTC* papillary thyroid carcinoma, *PTMC* papillary thyroid microcarcinoma, *HT* Hashimoto’s thyroiditis, *CLNM* central lymph node metastasis

*Numbers are presented as means ± standard deviation

Since both age and tumor size were the predictive factors of CLNM in PTC patients, it is necessary to try to find out the precise boundary that associated with CLNM. The relationship between CLNM and both patient age and tumor size was further analyzed using 10 years as an increment for age and 5 mm as an increment for tumor size (Table 3). It is interesting that all patients ≤ 30 years of age in the PTC > 10 mm group were positive for CLNM (100%, 51/51), and although the positivity rate decreased as age increased, it remained high at 60% (*P* < S0.001). In the PTMC group, the frequency of CLNM was 66% (29/44) in patients ≤ 30 years of age; however, it decreased dramatically with increasing age (*P* = 0.001). Regarding tumor size, the frequency of CLNM was only 31% (78/248) when the tumor size was ≤ 5 mm, and it increased with increasing tumor size. When the tumor size was > 20 mm, the rate of CLNM was greater than 80% (*P* < 0.001).

**Table 3:** Stratified analysis on the relationships between central lymph node metastasis and age, tumor diameter of PTC patients

Variable	Overall PTC(*n* = 943)					PTC > 10 mm(*n* = 373)					PTMC(*n* = 570)				
	Yes		No		*P*	Yes		No		*P*	Yes		No		*P*
	*n*	(%)	*n*	(%)		*n*	(%)	*n*	(%)		n	(%)	n	(%)	
Age (years)															
≤30	80	(84)	15	(16)	**<0.001**	51	(100)	0	(0)	**< 0.001**	29	(66)	15	(34)	**0.001**
30~	100	(56)	79	(44)		53	(79)	14	(21)		47	(42)	65	(58)	
40~	163	(49)	172	(51)		82	(66)	42	(34)		81	(38)	130	(61)	
50~	118	(49)	122	(51)		64	(70)	27	(30)		54	(36)	95	(64)	
50~	44	(47)	50	(53)		25	(63)	15	(37)		19	(35)	35	(65)	
Tumor diameter (mm)															
≤ 5	78	(31)	170	(69)	**< 0.001**	–	–	–	–		78	(31)	170	(69)	**<0.001**
5~	152	(47)	170	(53)		–	–	–	–		152	(47)	170	(53)	
10~	118	(67)	58	(33)		118	(67)	58	(33)	**0.02**	--	--	--	--	
15~	70	(76)	22	(24)		70	(76)	22	(24)		--	--	--	--	
> 20	87	(83)	18	(17)		87	(83)	18	(17)		--	--	--	--	

*Abbreviations*: *PTC* papillary thyroid carcinoma, *PTMC* papillary thyroid microcarcinoma

### The relationship between the *BRAF*^*V600E*^ mutation and the clinicopathological characteristics of PTC patients

The relationship between the *BRAF*^*V600E*^ mutation and the clinicopathological characteristics of the PTC patients are detailed in Table 4. In the overall PTC group, the incidence of *BRAF*^*V600E*^ mutation was statistically correlated with older age (*P* = 0.03), tumor size (*P* = 0.04), coexistent HT (*P* = 0.002), and early T stage (*P* = 0.02) but not with gender, multifocality, CLNM, or TNM stage. Multivariate analysis found that only age (*P <* 0.001, OR = 1.04, 95% CI 1.02–1.06) and coexistent HT (*P* = 0.005, OR = 0.35, 95% CI 0.17–0.73) were independent predictive factors of *BRAF*^*V600E*^ mutation status. In addition, both univariate and multivariate analyses uncovered that age (*P* = 0.004, OR = 1.03, 95% CI = 1.01–1.06) was a risk factor for *BRAF*^*V600E*^ mutation in the PTC > 10 mm group. Moreover, in the PTMC group, the *BRAF*^*V600E*^ mutation was significantly correlated with tumor size (*P* < 0.001*,* OR = 8.19, 95% CI = 2.73–24.57) and coexistent HT (*P* = 0.03*,* OR = 0.42, 95% CI = 0.19–0.92) by both univariate and multivariate analyses.

**Table 4:** Relationships between the *BRAF*^*V600E*^ mutation and clinicopathologic characteristics of 943 patients with PTC

Variable	The overall PTC					PTC > 10 mm group					PTMC				
	Positive	Negative	Uni- and multivariate analysis			Positive	Negative	Uni- and multivariate analysis			Positive	Negative	Uni- and multivariate analysis		
	*n*(%)	*n*(%)	*P*	*P*	OR(95CI)	*n*(%)	*n*(%)	*P*	*P*	OR(95CI)	*n*(%)	*n*(%)	*P*	*P*	OR(95CI)
Gender															
Male	199(85)	34(15)	0.974			86(80)	21(20)	0.324			113(90)	13(10)	0.287		
Female	607(86)	103(14)				225(85)	41(15)				382(86)	62(14)			
Age (years) ^*^	46.3 ± 11.8	44.0 ± 11.9	**0.03**	**< 0.001**	1.04 (1.02–1.06)	46.3 ± 11.7	41.0 ± 3.1	**0.002**	**0.004**	1.04 (1.01–1.06)	46.2 ± 10.9	46.6 ± 10.1	0.804		
Tumor diameter (mm) ^*^	1.1 ± 0.9	1.3 ± 1.2	**0.04**	0.283		1.9 ± 0.9	2.3 ± 1.2	**0.04**	0.756		0.6 ± 0.2	0.5 ± 0.2	**< 0.001**	**< 0.001**	8.19 (2.73–24.57)
Focality															
Uni	579(84)	109(16)	0.06			209(81)	50(19)	**0.039**	0.057		370(86)	59(14)	0.464		
Multi	227(89)	28(11)				102(90)	12(11)				125(89)	16(11)			
HT															
No	771(86)	122(14)	**0.002**	**0.005**	0.35 (0.17–0.73)	301(84)	57(16)	0.087			470(88)	65(12)	**0.007**	**0.03**	0.42 (0.19–0.92)
Yes	35(70)	15(30)				10(67)	5(33)				25(71)	10(29)			
CLNM															
No	379(86)	61(14)	0.588			87(89)	11(10)	0.095			292(85)	50(15)	0.206		
Yes	427(85)	76(15)				224(81)	51(19)				203(89)	25(11)			
T stage															
T1	724(86)	114(14)	**0.02**	0.683		229(85)	39(15)	0.088			495(87)	75(13)	–		
T2 + T3	82(78)	23(22)				82(78)	23(22)				–	–			
Tumor stage															
I	728(85)	128(15)	0.248			262(82)	58(18)	0.065			466(87)	70(13)	0.783		
II	78(90)	9(10)				49(93)	4(8)				29(85)	5(15)			

*Abbreviations*: *PTC* papillary thyroid carcinoma, *PTMC* papillary thyroid microcarcinoma, *HT* Hashimoto’s thyroiditis, *CLNM* central lymph node metastasis

*Numbers are presented as means ± standard deviation

The relationships between the *BRAF*^*V600E*^ mutation among age and tumor size were also further analyzed using 10 years as an increment for age and 5 mm as an increment for tumor size (Table 5). The *BRAF*^*V600E*^ mutation rate was maintained above 85% in the PTMC group, regardless of age. However, in the PTC > 10 mm group, the *BRAF*^*V600E*^ mutation rate was only 65% in patients ≤ 30 years of age (*P* = 0.003), and it rapidly increased to more than 85% in patients > 30 years of age. Furthermore, compared with tumor size ≤ 5 mm, the frequency of *BRAF*^*V600E*^ mutation was significantly higher (91%, 294/322) when the tumor size was in the 5–10 mm range (*P* < 0.001). The frequency of the *BRAF*^*V600E*^ mutation remained relatively high when the tumor size was > 10 mm.

**Table 5:** Stratified analysis on the relationships of *BRAF*^*V600E*^ mutation status with age and tumor size of PTC patients

Variable	Total PTC					PTC > 10 mm group					PTMC				
	Positive		Negative			Positive		Negative			Positive		Negative		
	*n*	(%)	*n*	(%)	*P*	*n*	(%)	*n*	(%)	*P*	*n*	(%)	*n*	(%)	*P*
Age															
≤ 30	72	(76)	23	(24)	0.08	33	(65)	18	(35)	**0.003**	39	(88)	5	(12)	0.818
30~	154	(86)	25	(14)		57	(85)	10	(15)		97	(87)	15	(13)	
40~	289	(86)	46	(14)		105	(85)	19	(15)		184	(87)	27	(13)	
50~	208	(87)	32	(13)		82	(90)	9	(10)		126	(85)	23	(15)	
60~	83	(88)	11	(12)		34	(85)	6	(15)		49	(91)	5	(9)	
Tumor size (mm)															
≤ 5	201	(81)	47	(19)	**0.001**	–	–	–	–		201	(81)	47	(19)	**< 0.001**
5~	294	(91)	28	(9)		–	–	–	–		294	(91)	28	(9)	
10~	152	(86)	24	(14)		152	(86)	24	(14)	0.197	–	–	–	–	
15~	77	(84)	15	(16)		77	(84)	15	(16)		–	–	–	–	
> 20	82	(78)	23	(22)		82	(78)	23	(22)		–	–	–	–	

*Abbreviations*: *PTC* papillary thyroid carcinoma, *PTMC* papillary thyroid microcarcinoma

### Relationships among CLNM, tumor size, and *BRAF*^*V600E*^ mutation status

The relationships among CLNM, tumor size, and *BRAF*^*V600E*^ mutation status are shown in Table 6. When the tumor size was ≤ 5 mm, the incidence of CLNM was significantly higher in *BRAF*^*V600E*^ mutation-positive patients than in mutation-negative patients (*P* = 0.009). When the tumor size was > 5 mm, there was no significant difference in CLNM rates between *BRAF*^*V600E*^ mutation-positive patients and mutation-negative patients.

**Table 6:** Relationships between central lymph node metastasis, tumor diameter, and the *BRAF*^*V600E*^ mutation status

Tumor diameter (mm)	*BRAF*^*V600E*^ mutation	Central lymph node metastasis						Statistic value	*P*
		Total		Yes		No			
		*n*	(%)	*n*	(%)	*n*	(%)		
≤ 5	Positive	210	(85)	72	(35)	138	(65)	6.822	**0.009**
	Negative	38	(15)	6	(16)	32	(84)		
5~	Positive	300	(93)	140	(47)	160	(53)	0.863	0.353
	Negative	22	(7)	12	(55)	10	(45)		
10~	Positive	311	(83)	224	(72)	87	(28)	2.549	0.110
	Negative	62	(17)	51	(82)	11	(18)		

## Discussion

Lymph node metastasis is commonly observed at diagnosis in PTC patients, even in PTMC patients. Although the *BRAF*^*V600E*^ mutation is the most common genetic event in PTC, its relationship with clinicopathological characteristics remains disputed. The purpose of this study was to analyze the predictive factors of both CLNM and the *BRAF*^*V600E*^ mutation in PTC patients.

In our study, the incidence of CLNM in all PTC patients was 53%, and it was significantly higher in the PTC > 10 mm group (74%) than in the PTMC group (40%). The CLNM prevalence ranged between 13.4% and 64% in previously reported studies [5–9]. This discrepancy may be due to the heterogeneity in surgeon proficiency, location of CLNM, and the precision of pathologists, because metastases are always prone to positive in lymph nodes with smaller size. CLNM is an important risk factor for PTC recurrence [5]; thus, it is necessary to identify the predictive factors associated with CLNM that may guide appropriate surgical strategies for patients.

Although the morbidity rate of male PTC patients was comparatively lower, the CLNM rate was significantly higher in male PTC patients (71%), which was in accordance with the findings of previously reported studies [14, 16], indicating that male might be a risk factor for CLNM in PTC patients. The normally higher basal metabolic rate of male patients might drive the overactive proliferation of tumor cells and lead to metastasis [5].

The results of this study also suggested that younger age is a risk factor for CLNM in PTC patients. The frequency of CLNM was significantly higher in PTC patients ≤ 30 years of age. The AJCC/UICC TNM system uses 45 years as the cutoff age for upstaging patients. Our results suggested 30 years might be a more appropriate cutoff age for predicting the risk for CLNM among Chinese patients. The results of a previous study indicated that the frequency of lymph node metastasis was 67% (64/95) in pediatric and young adult PTC patients (age ≤ 20 years) [17]. The correlation between age and CLNM needs to be further studied in a larger population.

Our results showed that the frequency of CLNM was dramatically increased when the tumor size was > 5 mm, which suggested that tumor size is a risk factor for CLNM. Larger tumor sizes contribute to more proliferative and aggressive tumors. Tumor size is known to be more frequently associated with CLNM [5, 15]. Although the current consensus states that tumor size > 1 cm is a risk factor for PTC, our results and those described in other reports [5, 16] indicate that CLNM is not uncommon among PTMC patients. Recently, an increasing number of PTMCs have been detected at early stages because of the use of advanced ultrasound technology. The relationship between CLNM and tumor size should be analyzed in a large-scale population to determine the tumor size that indicates the potential for the development of progressive PTC.

Our results suggested that coexistent HT in PTC patients is an independent protective factor against CLNM when the tumor size is > 10 mm. Some studies [18, 19] have revealed that coexisting HT with PTC is related to reduced nodal metastasis in comparison with that in patients without HT, and the presence of HT in PTC patients may protect against CLNM. However, other studies [5, 20] identified no difference in CLNM rates between PTC patients with or without HT. It is possible that the autoimmune response to thyroid-specific antigens in HT patients might be involved in the destruction of cancer cells expressing thyroid-specific antigen in PTC, thus preventing recurrence and LNM [18].

The incidence of *BRAF*^*V600E*^ mutation was 85% in the PTC patients, and the proportion was slightly higher in the PTMC group. Previous studies have shown that the *BRAF*^*V600E*^ mutation is common among PTC patients, and the overall prevalence varies from 40 to 78% among Chinese patients with PTC [5, 6, 21–24]. The possible reasons for the variation in positive mutation rates are PTC subtype, geographic region, ethnicity, and technical differences in the methods used to detect the *BRAF*^*V600E*^ mutation.

The results of the present study showed that age was an independent risk factor for the *BRAF*^*V600E*^ mutation. The *BRAF*^*V600E*^ mutation was common among PTMC patients, and the positivity rate was over 80%, regardless of age. Interestingly, in the PTC > 10mm group, the mutation rate was only 65% (33/51) in patients ≤ 30 years of age, but rapidly increased to 85%+ in patients > 30 years of age. Previous studies [6, 23] have also revealed an association between the *BRAF*^*V600E*^ mutation and elderly patients; however, no association was found in other studies [5, 9]. A possible reason for this phenomenon is that an increasing number of PTMCs are detected and treated at an early stage because of advanced examination methods. The *BRAF*^*V600E*^ mutation might occur early in thyroid carcinogenesis, and the effect might become more remarkable along with the development and progression of PTC [22].

In addition, our results showed that the *BRAF*^*V600E*^ mutation was more frequent in PTMC patients without HT compared with patients co-existent HT. Similar results were found in a meta-analysis [15] and other studies [5, 25]. In HT patients, T cells are abnormally activated and subsequently stimulate B cells to secrete a variety of autoantibodies [26]. Animal models [27] indicated that mice with thyroid-specific knock-in of oncogenic *BRAF* present invasive thyroid cancer and have high TSH levels. When these mice were crossed with TSH receptor knockout mice, the BRAF mutation was not able to induce cancer.

Our data showed that tumor size is an independent risk factor for *BRAF*^*V600E*^ mutation among PTMC patients. Although no significant difference was found between the *BRAF*^*V600E*^ mutation and the CLNM rate in PTC patients, stratified analysis revealed that the frequency of CLNM was significantly increased in *BRAF*^*V600E*^*-*mutated PTC patients when the tumor size was ≤ 5 mm. These results suggest that the *BRAF*^*V600E*^ mutation might play an important role in the carcinogenic process at an early stage. Many studies have revealed that CLNM is associated with the *BRAF*^*V600E*^ mutation [20–23], whereas other findings have indicated no relationship between these two factors [21, 24]. The inconsistent results might be because of the following factors: sample size, tumor subtypes, operative approaches, the total number of lymph node metastases removed, and the method used to detect the *BRAF*^*V600E*^ mutation.

The results of this study demonstrated that there were different characteristics between the PTC > 10 mm and PTMC groups. Distinctive analyses, including ultrasound, US-FNA biopsy, and clinical characteristics, should be put into practice before the treatment plan is determined. Not all PTC lesions are the same, and the lesion characteristics should be evaluated to determine which options are available for each patient [28]. Therefore, shared decision-making (SDM) should be recommended for clinical practice, which is an approach of care that involves the clinician using the best available evidence to discuss the attributes of different treatment options with patients to choose a method that makes intellectual and practical sense [29, 30].

There are several limitations in our study. First, this was a retrospective study, and the enrolled patients only represented a proportion of PTC patients to a certain extent. Second, the correlation of the *BRAF*^*V600E*^ mutation and progressive clinicopathological characteristics was restricted by the limited number of patients with advanced-stage disease. Currently, thyroid ultrasound examination is included in routine physical examinations in China because of the increased frequency of thyroid nodules. Third, the mean follow-up time was short in view of the natural course of thyroid cancer.

## Conclusions

In summary, the clinicopathological factors male, younger age (≤ 30 years), large tumor size (> 5 mm), and coexisting HT are independent predicative factors of CLNM. In addition, the BRAFV600E mutation is associated with large tumor size and absence of HT in PTMC patients and those aged > 30 years in the PTC > 10 mm group. Moreover, when the tumor size was ≤ 5 mm, the BRAFV600E mutation was an independent risk factor for CLNM. To realize optimal management, all these features should be comprehensively evaluated to design individualized regimens for PTC patients.

## Data Availability

The datasets used and/or analyzed during the current study are available from the corresponding author on reasonable request.

## Abbreviations

PTC: Papillary thyroid carcinoma
US–FNA: Ultrasonography-guided fine-needle aspiration
PTMC: Papillary thyroid microcarcinoma
*BRAF*: *B-type Raf kinase*
HT: Hashimoto’s thyroiditis
CLNM: Central lymph node metastasis
